# Suicidal attempts among psychiatric patients hospitalized in Tirana Psychiatric Service, Albania

**DOI:** 10.1192/j.eurpsy.2023.2376

**Published:** 2023-07-19

**Authors:** V. Alikaj, V. Skendi, E. Dashi

**Affiliations:** 1 Neuroscience department, Medical University, Neuroscience department, Medical University, Tirana, Albania

## Abstract

**Introduction:**

Suicide represents one of the most discussed mental health issues in the world today and health challenges for the future. The burden of suicide is calculated in very high numbers (800 thousand people per year) by the WHO (2014), ranking it among the other most frequent causes of death. The key criteria to determine suicidal or non-suicidal behavior is the presence of intentional self-injurious behavior, in which the individual intentionally attempts to harm himself.

**Objectives: Aim of the study:** was to make a presentation and evaluation of the demographic and clinical factors of suicidal self-injurious behavior in patients hospitalized

**Methods:**

Patients’ data were obtained from the archived clinical files of the Psychiatric Emergency Department and other wards at “Xhavit Gjata” Tirana Psychiatric Hospital. The method used is a descriptive retrospective study of patients admitted during period of January-May 2019. About 75 archived clinical files were thoroughly studied and analyzed, on various demographic and clinical variables.

**Results:**

Albanian women remain more at risk for suicide attempts, while male suicide mortality is higher, as in the world. Higher determination and the use of more lethal methods ranked among the factors contributing to the higher mortality of men. The 29-49 age group is the most affected in our study according to suicide attempts and self-harming behavior. The most pronounced accompanying diagnoses of suicide attempts are major depressive disorders, but not leaving behind psychotic disorders. The average length of stay in the hospital is 18.4 days.

**Conclusions:**

The deepening of knowledge on the etiology, on the factors influencing suicide and on the methods of treatment are only some of the issues facing public health today. Identification of self-injurious behaviors pave the way for treatment and assistance for anyone considering suicide.

**Disclosure of Interest:**

None Declared

